# Functional Connectivity of the Precuneus in Female University Students with Long-Term Musical Training

**DOI:** 10.3389/fnhum.2016.00328

**Published:** 2016-06-29

**Authors:** Shoji Tanaka, Eiji Kirino

**Affiliations:** ^1^Department of Information and Communication Sciences, Sophia UniversityTokyo, Japan; ^2^Department of Psychiatry, Juntendo University School of MedicineTokyo, Japan; ^3^Juntendo Shizuoka HospitalShizuoka, Japan

**Keywords:** default mode network, fMRI, insula, mental imagery, music, operculum, performance, resting state

## Abstract

Conceiving concrete mental imagery is critical for skillful musical expression and performance. The precuneus, a core component of the default mode network (DMN), is a hub of mental image processing that participates in functions such as episodic memory retrieval and imagining future events. The precuneus connects with many brain regions in the frontal, parietal, temporal, and occipital cortices. The aim of this study was to examine the effects of long-term musical training on the resting-state functional connectivity of the precuneus. Our hypothesis was that the functional connectivity of the precuneus is altered in musicians. We analyzed the functional connectivity of the precuneus using resting-state functional magnetic resonance imaging (fMRI) data recorded in female university students majoring in music and nonmusic disciplines. The results show that the music students had higher functional connectivity of the precuneus with opercular/insular regions, which are associated with interoceptive and emotional processing; Heschl’s gyrus (HG) and the planum temporale (PT), which process complex tonal information; and the lateral occipital cortex (LOC), which processes visual information. Connectivity of the precuneus within the DMN did not differ between the two groups. Our finding suggests that functional connections between the precuneus and the regions outside of the DMN play an important role in musical performance. We propose that a neural network linking the precuneus with these regions contributes to translate mental imagery into information relevant to musical performance.

## Introduction

Mental imagery plays a role in musical processing for both performers and listeners (Herholz et al., 2012; Keller, 2012; Beaty et al., 2013). Musicians deliberately use concrete mental imagery to improve musical expression and performance. Mental imagery and emotion are tightly linked; music evokes emotions associated with conceived mental imagery that are qualitatively similar to emotions experienced during everyday life (Juslin and Västfjäll, 2008). Emotion is so critical to music that musicians are trained to create mental imagery for each piece of music and to reflect these images in their performance. However, the neural substrates of the transformational process from mental imagery into musical performance have not yet been elucidated.

To some extent, the creation of mental imagery, or scene construction, is constrained by or dependent upon episodic memory: the memory of personal experiences, including times and places, which are linked with emotions and other contextual information (Dickerson and Eichenbaum, 2010). Episodic memory retrieval has been suggested to be a reconstructive process involving the synthesis of various mental images related to a memory (Hassabis and Maguire, 2007, 2009; Hassabis et al., 2007). The episodic memory system is highly adaptive (Schacter et al., 2012) and thus plays a critical role in formulating appropriate behaviors in a continuously changing environment. The adaptive and constructive nature of episodic memory is highly relevant to musical performance because the mental scene progresses continuously throughout a performance. Previous studies show that visual imagery and scene-construction tasks activate a variety of brain regions, including the prefrontal cortex, pre-supplementary motor area, precuneus, angular gyrus (AG), occipital cortex, and hippocampus (Mechelli et al., 2004; Hassabis and Maguire, 2009; Summerfield et al., 2010). Mechelli et al. (2004) demonstrated increased connectivity between prefrontal and visual cortical areas during visual imagery compared to that during visual perception tasks. Therefore, mental image construction is cooperatively mediated by multiple brain regions (Mechelli et al., 2004).

The default mode network (DMN), whose core is composed of the medial prefrontal cortex (mPFC), posterior cingulate cortex (PCC), precuneus/PCC, and bilateral AG, has been hypothesized to generate spontaneous thoughts during mind-wandering and is believed to play an essential role in creativity (Buckner et al., 2008; Vessel et al., 2012, 2013; Utevsky et al., 2014). To date, a variety of functions have been associated with this network; however, its overall function remains controversial (Seghier and Price, 2012). The DMN was originally thought to be deactivated during externally assigned tasks (Raichle et al., 2001). However, more recent studies have demonstrated that the DMN is in fact activated during certain tasks (Spreng, 2012; Vatansever et al., 2015a,b). The involvement of the DMN in goal-directed cognitive tasks is controversial. One study found that the DMN was deactivated during attention and working memory tasks (Mayer et al., 2010), while other studies have demonstrated dynamic changes in functional connectivity between the DMN and task-related somatomotor regions during a finger opposition task (Vatansever et al., 2015a,b). DMN activation has been observed during tasks that involve episodic memory retrieval, envisioning future events, inferring the thoughts and perspectives of others, and social cognition (Addis et al., 2007; Buckner et al., 2008; Mars et al., 2012; Moran et al., 2012). All of these tasks involve scene-construction processes (Hassabis and Maguire, 2009), suggesting overlap between the DMN and neural networks that underlie scene construction. Interestingly, the DMN is activated by music as well as other forms of art (Vessel et al., 2012, 2013), supporting the association of scene construction processes with music and other arts.

Located in the posterior part of the cortical midline structures, the precuneus and surrounding areas show the highest resting metabolic rates among all brain structures (Cavanna and Trimble, 2006). The precuneus is one of the core nodes of the DMN (Cavanna and Trimble, 2006; Fransson and Marrelec, 2008; Utevsky et al., 2014) and shows widespread functional connectivity across the entire cortical area (Margulies et al., 2009; Zhang and Li, 2012). Importantly, the precuneus plays a critical role in mental image processing by integrating multimodal information collected from a wide variety of brain regions (Hassabis and Maguire, 2009; Summerfield et al., 2010). Graph theoretical analyses have suggested that the precuneus is 1 of 12 “rich-club nodes” that are highly connected to other brain regions and to one another (van den Heuvel and Sporns, 2011, 2013; Ottet et al., 2013). Furthermore, dynamic causal modeling studies have suggested that the precuneus plays a central role in the visual imagery network (Mechelli et al., 2004). The precuneus also plays a pivotal role in constructive processing, which is important for multiple functions such as predicting future occurrences and judging fitness of an object or tool for a particular purpose (Hassabis and Maguire, 2007, 2009). A previous study that compared playing the piano in the mind with actual performance demonstrated that the precuneus was activated under both conditions (Meister et al., 2004), indicating that the integrative mental image processing capability of the precuneus contributes to musical performance.

Musicians would unconsciously utilize constructive processes when creating mental imagery during performance that enable them to visualize the entire course of a performance and to create sounds that represent his or her mental images. Therefore, we predicted that the functional connectivity of the precuneus is altered in musicians compared to nonmusicians. In this study, we used functional magnetic resonance imaging (fMRI) to investigate the functional network that mediates the conversion of mental imagery to an internal representation that is used during musical performance. Because the precuneus is critically involved in the creation and processing of mental imagery, this structure would be central to the network. We performed resting-state fMRI in female university students majoring in music and nonmusic disciplines to examine whether there is altered connectivity of the precuneus in musicians.

## Materials and Methods

### Participants

Study procedures were approved by the Ethics Committees of Sophia University and Juntendo University. University students majoring in music were recruited for this study using advertisements (*n* = 26; age, 18−27 years; mean age, 21.5 years) and nonmusic disciplines (*n* = 26; age, 19−27 years; mean age, 21.3 years). All participants were healthy, right-handed Japanese females with no history of neurological or neuropsychiatric disorders. Among the students majoring in music, musical training had begun at 3−5 years of age and continued through the start of the present study. These students specialized in classical music played on various instruments (piano, violin, cello, contrabass, clarinet, or trumpet). The majors of nonmusicians were diverse, including literature, philosophy, psychology, economics, and science and engineering. All subjects provided written informed consent before participation in the study. All subjects completed the imaging and the data were used for the analysis of this study.

### Image Acquisition and Preprocessing

Blood-oxygen-level dependent (BOLD) fMRI data were collected during a resting-state session with the eyes closed. Data were acquired using a Philips Achieva 3.0 Tesla MRI scanner at Juntendo University Hospital. A T2*-weighted gradient-echo echo-planar imaging (EPI) sequence was used with the following parameters: echo time (TE) = 30 ms; repetition time (TR) = 2000 ms; field of view (FOV) = 240 × 240 mm; matrix = 64 × 64; flip angle = 90°; number of axial slices = 33; voxel size = 3.75 × 3.75 × 4.00 mm. Each session consisted of 200 scans (total time, 6 min 40 s). Imaging data were preprocessed using the CONN toolbox (Whitfield-Gabrieli and Nieto-Castanon, 2012) running on MATLAB (version 8.3.0, MathWorks Inc., 2014). The first four volumes were discarded, and the remaining 196 volumes were subjected to preprocessing: The slice timing was corrected according to the slice order. The fMRI data were realigned and subsequently normalized to the standard Montreal Neurological Institute (MNI) template as implemented in the Statistical Parametric Mapping (SPM) Software platform. Image artifacts originating from head movement were handled using the ART scrubbing procedure^1^. Signal contributions from white brain matter, cerebrospinal fluid, and micro head-movement (six parameters) were regressed out from the data. The fMRI data were bandpass filtered (0.008–0.09 Hz). All functional images were spatially smoothed using a Gaussian filter kernel (full width at half maximum, FWHM = 8 mm).

### Data Analysis

Functional connectivity analysis was performed using the CONN toolbox. In individual analysis, Pearson’s correlation coefficients were calculated between the time course of the precuneus, defined by the Harvard-Oxford Atlas, and the time courses of all other voxels, which provided a seed-to-voxel connectivity matrix. Positive and negative correlation coefficients defined positive and negative functional connectivity, respectively (Whitfield-Gabrieli and Nieto-Castanon, 2012). The correlation coefficients were then converted to normally distributed scores using Fisher’s transform, which were subsequently used in the population-level analysis. The connectivity matrix with converted scores was compared between the music and nonmusic groups. The hight-threshold of *p* < 0.001, uncorrected, was applied to individual voxels to define clusters. The extracted clusters were thresholded at *p* < 0.05 with the false discovery rate (FDR) correction to report the results.

## Results

**Figure 1:**
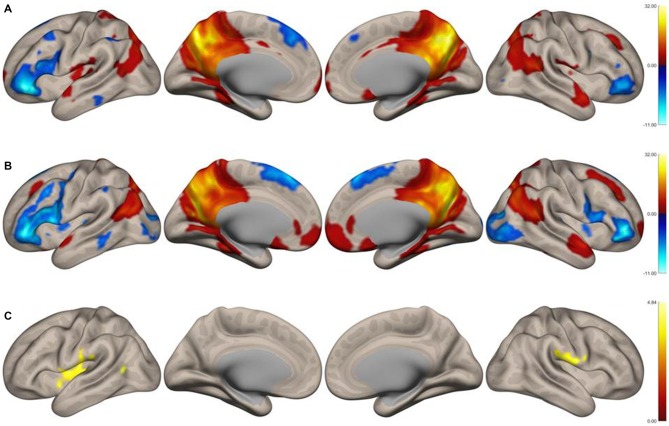
**Surface functional connectivity maps of the precuneus as the seed region in musicians. (A)** Music group, **(B)** nonmusic group, **(C)** music vs. nonmusic. The maps were thresholded at *p* < 0.05, false discovery rate (FDR)-corrected.

Functional connectivity maps of the precuneus are shown in Figures 1A,B. Regions of negative functional connectivity appeared larger in nonmusicians than in musicians. Musicians showed connectivity between the precuneus and insular cortex (IC)/central operculum (CO)/parietal operculum (PO), Heschl’s gyrus (HG)/planum temporale (PT), and the left inferior lateral occipital cortex (iLOC). Figure 1C shows regions with significantly higher connectivity to the precuneus in musicians compared to nonmusicians. These regions were composed of three clusters of voxels, which are summarized in Table 1. Connectivity of the precuneus with other structures of the DMN did not differ between the groups.

**Table 1:** **Functional connectivity of the precuneus as the seed region that was higher in musicians compared to nonmusicians**.

Clusters (*x, y, z*)	Voxel size	Cluster *p* (FDR)
Cluster 1 (−34 −34 +08)	1037	0.000
Cluster 2 (+60 −30 +22)	542	0.000
Cluster 3 (−56 −64 +08)	188	0.029
**Cluster 1**
330 voxels covering 34% of CO.L
221 voxels covering 17% of IC.L
142 voxels covering 46% of HG.L
82 voxels covering 15% of PT.L
68 voxels covering 12% of PO.L
**Cluster 2**
147 voxels covering 27% of PO.R
96 voxels covering 3% of PostCG.R
75 voxels covering 9% of CO.R
53 voxels covering 12% of PT.R
**Cluster 3**
142 voxels covering 7% of iLOC.L

*There were no regions showing significantly lower connectivity in musicians compared to nonmusicians. CO, central operculum; IC, insular cortex; HG, Heschl’s gyrus; PT, planum temporale; PO, parietal operculum; PostCG, post-central gyrus; iLOC, lateral occipital cortex, inferior division*.

## Discussion

In this study, we analyzed the functional connectivity of the precuneus in female musicians and nonmusicians. Connectivity strength between the precuneus and the opercular/insular regions, HG/PT regions, and LOC differed between the two groups. None of the regions within the DMN showed different connectivity with the precuneus between the groups.

### Roles of the Insula and Operculum in Musical Performance and Sensory Integration

Musicians showed higher connectivity between the precuneus and the insula, a cortical region that links external and internal information processing (Lamm and Singer, 2010; Couto et al., 2013). Because it is highly connected with limbic, sensory, and motor regions, the insula is involved in the processing of emotional, sensorimotor, visceral, and interoceptive information (Craig, 2009). In particular, the insula is critically involved in evaluating the emotional salience of both external and interoceptive stimuli (Craig, 2009; Taylor et al., 2009; Menon and Uddin, 2010; Straube and Miltner, 2011; Couto et al., 2013). Together with the dorsal cingulate cortex, the insula constitutes the core part of a salience network that integrates cognitive and emotional information to make appropriate responses (Menon, 2015), which is highly relevant to musical performance. The insula also participates in emotional perception and social cognition (Jabbi et al., 2008; Menon and Uddin, 2010); this function is linked to the role of the insula in interoception, which is thought to be important for understanding the emotional states of oneself and others (Singer et al., 2004; Straube and Miltner, 2011; Seth, 2013; Ondobaka et al., 2015). The insula has been implicated in emotional responses to music (Blood and Zatorre, 2001; Brown et al., 2004; Griffiths et al., 2004; Trost et al., 2012).

The central and posterior operculum showed higher connectivity with the precuneus in musicians. Previous studies have demonstrated activation of the operculum while performing or listening to music. For example, the operculum is activated in opera singers during actual and imagined singing of an Italian aria (Kleber et al., 2007) and in subjects listening to pleasant music (Koelsch et al., 2006). The operculum and insula are continuous and interconnected structures and appear to work cooperatively. The CO and insula have been suggested to be involved in music-related emotional processing (Gebauer et al., 2014), consistent with the results of a previous study showing that a patient with a CO/insular lesion was unable to experience emotional responses to music (Griffiths et al., 2004). A recent stepwise functional connectivity analysis showed that functional connections from sensory areas converge on the operculum/insula, suggesting that this region is involved in the integration of multimodal sensory information and may form a connection between auditory and somatosensory areas (Sepulcre et al., 2012). Further, the OP4 region of the PO is connected to both the auditory and motor cortices and may therefore play a critical role in audiomotor integration (Sepulcre, 2015). Therefore, a neural network connecting the precuneus with the operculum and insula may integrate mental imagery with interoceptive and emotional information to influence musical performance. In our analysis, higher precuneus connectivity in the left hemisphere of musicians was detected by a larger cluster in the CO than in the PO. In the right hemisphere, however, the cluster in the PO was larger than that in the CO. This left-lateralized connectivity between the precuneus and the CO may be due to the frequent dominance of the right hand when playing an instrument such as the piano. Similarly, when playing a string instrument such as the violin or cello, bowing with the right hand is a significant factor in the emotional expression of the music.

### Heschl’s Gyrus and the Planum Temporale

Functional connectivity between the precuneus and the HG/PT region was also higher in musicians compared to nonmusicians. The HG/PT region processes music-related pitch information (Hall and Plack, 2009; Puschmann et al., 2010; Angulo-Perkins et al., 2014). Voxel-based morphometry studies have reported that the HG is larger in musicians than in nonmusicians (Gaser and Schlaug, 2003; Bermudez et al., 2009). The PT, which partially overlaps with Wernicke’s area, is a critical region for the processing of various aspects of sound and is involved in the analysis of sounds with complex spectrotemporal structure (Griffiths and Warren, 2002). Therefore, connectivity between the precuneus and the HG/PT region may allow the integration of mental imagery with analysis of complex sounds; the higher connectivity between these regions in musicians may reflect the fact that musicians are trained to integrate imagery and sound information. Moreover, the HG/PT region has been demonstrated to have strong connections with the operculum (Sepulcre et al., 2012; Sepulcre, 2015), suggesting further integration of sounds with emotional, interoceptive, and sensorimotor information.

### The Lateral Occipital Cortex

Musicians also showed higher connectivity between the precuneus and the left LOC compared to nonmusicians. The LOC plays an important role in object perception (Malach et al., 2002; Nagy et al., 2012). An fMRI study has suggested that this region also participates in scene construction from objects (MacEvoy and Epstein, 2011). Therefore, the observed increase in connectivity between the precuneus and LOC further supports our hypothesis that musicians utilize scene construction during musical performance.

### Limitations of the Study

Resting-state functional connectivity analysis is suited for exploring differences in connectivity between musicians and nonmusicians. However, the causal relationship between the observed group differences and musical training cannot be assessed in this cross-sectional study. For the test of the causal relationship, a longitudinal study is necessary. There is another uncertainty that the part of network whose differences in connectivity have been detected in this study is truly utilized in mental imagery processing for musical performance. To verify this, an fMRI experiment with “imagined” musical performance is currently in progress. By combining the results from the resting-state and task-fMRI, one will be able to associate the alteration of functional connectivity in musicians with mental imagery processing for musical performance.

## Conclusion

The precuneus has been associated with visuospatial and motor imagery, episodic and autobiographical memory retrieval, and self-related information processing (Cavanna and Trimble, 2006; Zhang and Li, 2012). Creating mental imagery requires the integration of various types of information, consistent with findings that the precuneus is connected to many cortical and subcortical areas (Cavanna and Trimble, 2006) and is involved in the multistep functional convergence of visual, auditory, and somatosensory information (Sepulcre et al., 2012). Thus, the precuneus is a connector hub that communicates with many brain regions and is a central site for the creation of mental imagery. We demonstrate that musicians show higher connectivity between the precuneus and brain regions involved in the processing of auditory, interoceptive, sensorimotor, and emotional information, indicating that the precuneus plays an important role in musical performance. We propose that this higher connectivity contributes to the translation of mental imagery into information to be utilized by the motor control system during musical expression and performance.

## Author Contributions

ST and EK planned and conducted all the experiments. ST analyzed the data and wrote the manuscript.

## Conflict of Interest Statement

The authors declare that the research was conducted in the absence of any commercial or financial relationships that could be construed as a potential conflict of interest.

## Footnotes

^1^www.nitrc.org/projects/artifact_detect/
